# The Impact of Endometrioma on Embryo Quality in In Vitro Fertilization: A Retrospective Cohort Study

**DOI:** 10.3390/jcm12062416

**Published:** 2023-03-21

**Authors:** Houjin Dongye, Yizheng Tian, Dan Qi, Yanbo Du, Lei Yan

**Affiliations:** 1 Center for Reproductive Medicine, Shandong University, Jinan 250012, China; 2 Key Laboratory of Reproductive Endocrinology of Ministry of Education, Shandong University, Jinan 250012, China; 3 Shandong Key Laboratory of Reproductive Medicine, Jinan 250012, China; 4 Shandong Provincial Clinical Research Center for Reproductive Health, Jinan 250012, China; 5 National Research Center for Assisted Reproductive Technology and Reproductive Genetics, Shandong University, Jinan 250012, China

**Keywords:** endometrioma, oocyte quality, embryo quality, IVF

## Abstract

The influence of endometrioma on oocyte and embryo competence is inconclusive. Furthermore, the benefits of surgical treatment remain uncertain. This study aimed to investigate the effect of endometrioma on oocyte and embryo quality from a morphological perspective and further explore whether surgery could contribute to improving oocyte and embryo competence. A total of 664 IVF cycles with endometrioma (538 cycles underwent surgeries) and 3133 IVF cycles from the control group were included. The propensity score matching was used to balance the baseline differences between groups. There was a lower MII oocyte rate (85.0% versus 87.8%, *p* < 0.001; 84.9% versus 87.6%, *p* = 0.001) and a similar good-quality embryos rate in women with endometrioma (and those who underwent surgeries) compared with control group. For women with endometrioma, the rates of blastocyst development (67.1% versus 60.2%; *p* = 0.013) and good blastocyst development (40.7% versus 35.2%; *p* = 0.049) were significantly higher in those who had undergone surgical treatment compared with those who had not, but the rates of MII oocytes (79.9% versus 87.7%; *p* < 0.001) and normal fertilization (55.2% versus 66.2%; *p* < 0.001) were lower. The study indicates that endometrioma, including its surgical treatment, compromises the oocyte maturity not the embryo quality at the cleavage stage; however, the surgery seems to contribute to improving blastocyst development.

## 1. Introduction

Endometriosis is a common gynecological disease characterized by the presence of endometrial glands and stroma outside the uterine cavity, increasingly considered a chronic inflammatory condition [1,2]. This affects 5–10% of reproductive aged women and up to 50% of infertile women [3,4]. Endometrioma, the most common pathotype of endometriosis, is present at 17–44% of patients [5]. Numerous studies have demonstrated the negative effect of endometriosis, especially endometrioma, on female fertility [6,7,8]. It is estimated that 30–50% of patients are afflicted with infertility [9]. A meta-analysis by Harb et al., pooling the results from 27 observational studies, a total of 8984 patients, showed a lower implantation rate and clinical pregnancy rate in women with stage III/IV endometriosis compared with women without endometriosis [10]. Opøien et al. reported a significantly decreased live birth rate in women with endometriomas by classifying women with stage III/IV endometriosis into groups with and without endometriomas [11]. However, the exact pathogenic mechanisms of endometrioma-related infertility remain unclear. Several factors have been proposed to account for this problem such as distorted tubo-ovarian anatomy, mechanical stretching, alteration in follicular microenvironment, impaired endometrial receptivity, chronic inflammatory changes in the pelvic cavity, and reduced oocyte and embryo competence [12,13,14,15].

It is already acknowledged that oocyte and embryo quality is vital to a successful outcome of in vitro fertilization (IVF). At present, a number of criteria for evaluating oocyte and embryo quality mainly based on morphological characteristics have been established to select high-quality oocytes and embryos for improving subsequent pregnancy outcomes. In recent years, the issue that the influence of endometrioma on oocyte and embryo competence has raised growing attention; nevertheless, the results of these studies are inconclusive. González-Foruria et al. indicated that the number of oocytes retrieved and metaphase stage II (MII) oocytes was lower in 101 women with endometriomas in comparison with 822 women with infertility factors other than endometriosis [7]. Conversely, Reinblatt et al. failed to find this significant difference in the number of retrieved oocytes and MII oocytes between women with bilateral endometriomas and those who had undergone IVF due to tubal or malefactor infertility [16]. Moreover, Filippi et al. conducted a prospective cohort study, and the result showed the oocytes quality and the rate of high-quality embryos were comparable between affected and intact ovaries in women with unoperated unilateral endometrioma [17]. Surgery is the major treatment modality for endometrioma when intervention is required, however, the benefits of surgery remain uncertain, including whether surgery would damage an ovarian reserve or improve oocyte and embryo competence.

This study aims to investigate the effect of endometrioma on oocyte and embryo quality from the morphological perspective and further explore whether surgery could contribute to improving the oocyte and embryo competence.

## 2. Materials and Methods

### 2.1. Study Design and Population

This retrospective cohort study analyzed IVF data from the Reproductive Hospital Affiliated to Shandong University between January 2013 and December 2019. The study population was patients with endometrioma diagnosed by ultrasonography. All patients from the endometrioma with surgery group had undergone cystectomy by laparoscopy or laparotomy. The control group consisted of women with infertility due to tubal factors during the same period. The inclusion criteria were as follows: age ≤ 40 years; women without non-endometriotic ovarian cyst; control population had not undergone surgery for ovary; normal sperm (concentration ≥ 15 × 10^6^/mL, total motility ≥ 40%, normal morphology ≥ 4%) in the male according to the fifth edition of World Health Organization (WHO) guidelines. The exclusion criteria included: intracytoplasmic sperm injection (ICSI), preimplantation genetic testing (PGT), hydrosalpinx, pelvic adhesions, polycystic ovarian syndrome (PCOS), primary ovarian insufficiency (POI), premature ovarian failure (POF), decreased ovarian reserve, hyperprolactinemia, hyperthyroidism, hypothyroidism, adrenal disease, adenomyosis, and cycles with donated oocytes or sperm. This research was approved by the Institutional Review Board (IRB) of Reproductive Hospital affiliated to Shandong University (2020-14).

### 2.2. IVF Procedures

Controlled ovarian hyperstimulation, oocyte retrieval, fertilization, embryo culture, and evaluation were in line with our center’s standard protocols as previously reported [18]. In brief, the ovarian stimulation protocol was determined based on the patient’s infertility cause, ovarian function, age, and menstrual cycle. Several commonly used stimulation protocols included long gonadotropin releasing hormone (GnRH) agonist protocol, short GnRH agonist protocol, ultra-long GnRH agonist protocol, and GnRH antagonist protocol, whilst other unconventional protocols included a mild stimulation protocol and natural cycle protocol, but the above stimulation protocols were previously described in detail [19]. The gonadotropin dose was adjusted depending on the follicular growth monitored by transvaginal ultrasound (TVUS) scan and serum sex steroids tests. In our hospital, recombinant follicle stimulating hormone was administered during controlled ovarian hyperstimulation and human menopausal gonadotropin could be added at the discretion of different doctors. The final oocyte maturation was triggered with human chorionic gonadotropin (hCG) at a dose of 4000–10,000 IU when at least two follicles measured 18 mm or more in mean diameter. Oocyte retrieval guided by TVUS was performed 34–36 h after hCG administration. IVF was carried out according to the semen parameters approximately 4–6 h after follicular aspiration. Embryo development was assessed by morphologic criteria at our center. The cleavage stage embryos were scored by Puissant criteria on the basis of the number and size of blastomeres as well as the percentage of anucleate fragments [20]. The blastocysts were graded by Gardner criteria on the basis of the degree of blastocyst expansion as well as the development of the inner cell mass (ICM) and trophectoderm (TE) [21]. Fresh embryo transfer could be cancelled in some cases such as ovarian hyperstimulation syndrome (OHSS), early elevated progesterone level, no viable oocytes or embryos, and thin endometrium.

### 2.3. Study Outcomes

The primary outcome was a good-quality embryo rate which was defined as the proportion of good-quality embryos over normally fertilized oocytes. The good-quality embryos were defined as 7–10 cells without multinucleation, ≥3 points, and cultured from normal zygotes on Day 3. The second outcomes were the number of oocytes retrieved, rates of MII oocytes, normal fertilization, embryo development (Day2, Day3), blastocyst development, and good blastocyst development. The good blastocysts were considered embryos with an expansion score ≥ 3, without C in the development of ICM and TE on Day 5 and Day 6. The detailed definitions of the above study outcomes were in accordance with the Vienna consensus [22].

### 2.4. Statistical Analysis

The normality of data was assessed using the Shapiro–Wilk test. Continuous variables were represented as median (interquartile range), with the Mann–Whitney U test for between-group differences. Categorical data were expressed as frequency and percentage, and the differences between groups were examined via Pearson chi-square test or Fisher’s exact test. A *p* value < 0.05 was considered statistically significant. To reduce the impact of selection bias and confounding factors, propensity score matching (PSM) was used. The propensity score model was built using the multivariable logistic regression analysis that included all baseline characteristics. A 1:1 matching was performed using nearest neighbor matching with a caliper width of 0.02, and without replacement. The standardized mean difference (SMD) was calculated to assess the between-group balance of baseline characteristics before and after matching. An absolute value of SMD less than 0.1 was interpreted as comparability. All statistical analyses were conducted with the use of R programming language (version 4.1.2, R Core Team 2021, Vienna, Austria) and Statistical Package for the Social Sciences (SPSS) software (version 26.0, IBM, Chicago, IL, USA).

## 3. Results

### 3.1. Basal Characteristics between Groups

A total of 664 IVF cycles with endometrioma, among which 538 cycles underwent endometrioma-related surgeries, and 3133 IVF cycles from control group were included in the analysis. No differences were observed only in sperm concentration and total motility, the proportion of short GnRH agonist protocol, days of ovarian stimulation, and gonadotrophin initiating dose of all 19 baseline variables between endometrioma and control group. After matching, 532 pairs of cycles were included, and all baseline characteristics were comparable between groups (Table 1). For subgroup analysis, before matching, the absolute value of SMD for all baseline variables was more than 0.1 except for sperm concentration and total motility, the proportion of short GnRH agonist protocol, days of ovarian stimulation, gonadotrophin initiating dose, and endometrial thickness on the hCG trigger day between the endometrioma with surgery and control groups. After matching, the two comparison groups were balanced, and 441 pairs of cycles were included (Table S1). For endometrioma with and without surgery, before matching, there were significant differences in the 13 baseline characteristics between endometrioma with and without surgery. After matching, 109 pairs of cycles were included, and the between-group differences were not significant (Table S2).

### 3.2. Outcomes

The MII oocytes rate was significantly lower in the endometrioma group than in the control group (85.0% versus 87.8%; *p* < 0.001). Additionally, there was a trend toward a lower good-quality embryos rate in the endometrioma group compared with control group, although the difference did not reach statistical significance. In addition, other outcomes such as the number of oocytes retrieved, the rates of normal fertilization, embryo development (Day 2, Day 3), blastocyst development, and good blastocyst development were similar between the two groups. There were no significant differences in the rates of clinical pregnancy, miscarriage, and live birth (Table 2).

The good-quality embryos rate appeared to be higher in endometrioma with surgery than in the control group, however, the difference was not statistically significant. The cycles of endometrioma with surgery was associated with lower MII oocytes rate than the control group (84.9% versus 87.6%; *p* = 0.001). Beyond that, other outcomes including pregnancy outcomes did not differ significantly between comparison groups (Table 3).

For endometrioma with and without surgery, the MII oocytes rate was significantly lower in the endometrioma group with surgery (79.9% versus 87.7%; *p* < 0.001), while the good-quality embryos rate was comparable between groups. The surgery for endometrioma may result in a lower normal fertilization rate (55.2% versus 66.2%; *p* < 0.001). Nevertheless, there were higher rates of blastocyst development and good blastocyst development in the endometrioma group with surgery (67.1% versus 60.2%, *p* = 0.013; 40.7% versus 35.2%, *p* = 0.049). As for other outcomes including pregnancy outcomes, no significant between-group differences were found (Table 4).

## 4. Discussion

The study demonstrated that there was a lower MII oocytes rate and a similar good-quality embryos rate in women with endometrioma (as well as those who underwent surgical treatment) compared with the control group, other outcomes were comparable. For women with endometrioma, the rates of blastocyst development and good blastocyst development were significantly higher in those that had prior surgical treatment compared with those who had not; however, the rates of normal fertilization and MII oocytes were lower. Moreover, other outcomes including good-quality embryos rate did not significantly differ between the two groups.

A growing number of cohort studies have investigated the effect of endometrioma on oocyte and embryo quality in IVF/ICSI, but there was substantial heterogeneity across these studies whether in study design or results. Benaglia et al. included 39 women with unoperated bilateral endometriomas and 78 control subjects by 1:2 matching, and reported a comparable number of high-quality embryos but a significantly lower number of oocytes retrieved and suitable oocytes which included MII oocytes and type 1 cumulus-oocyte complex in the endometriomas group [23]. Similarly, Suzuki et al. failed to detect a difference in the rate of good-quality embryos between 80 IVF cycles with endometrioma and 283 cycles with tubal factor infertility, and they found a lower number of oocytes retrieved in the study group, but the MII oocytes rate was not shown [24]. Notably, these studies were all small sample sizes. In contrast, a large retrospective cohort study conducted by Wu et al. indicated a significantly lower number of top-quality embryos and blastocyst rates, in addition to a decreased number of oocytes retrieved and oocyte maturation rate in endometrioma group compared with control group [25]. Furthermore, a meta-analysis by Yang et al., pooling the results from nine studies, reported a similar number of good-quality embryos but a lower number of MII oocytes in endometrioma group, they also made comparisons in unilateral endometrioma, but no difference was found in the number of MII oocytes and embryos formed between ovaries affected and contralateral normal ovaries [26]. There are many underlying mechanisms for the negative effect of endometrioma on oocytes quality. It has been proposed that the altered follicular microenvironment such as excessive reactive oxygen species and free radicals production and high expression of inflammatory cytokines would lead to DNA damage and meiotic spindle disorganization; thus, the oocytes quality is compromised [27,28,29]. However, the exact pathophysiology remains to be elucidated.

The surgical management of endometrioma prior to IVF/ICSI is a point deserving attention, with increasing evidence questioning the benefits of surgery. More recent studies have observed the decreased ovarian reserve after cystectomy, and the damage may be due to the removal of the normal ovarian tissue or electrocoagulation injury during operation [30,31]. Our research indicated that surgery for endometrioma had no negative impact on embryos quality on Day 3, but was associated with compromised oocytes maturity. Furthermore, the blastocysts quality was found to be improved in this population, and this finding would contribute to patient counselling and clinical practice as the blastocyst-stage embryo transfer—especially the frozen single blastocyst transfer—strategy is becoming increasingly popular [32]. In line with our results, Li et al. reported a comparable high-quality embryo rate per oocyte retrieved and a lower number of MII oocytes in women with endometrioma having undergone cystectomy compared with those who underwent aspiration, whereas the difference in the viable blastocyst rate was not observed [33]. At present, only one randomized controlled trial on this issue was published, enrolling a total of 99 women with endometriomas measuring 3–6 cm in diameter, which demonstrated that surgery for endometrioma resulted in a reduced number of retrieved mature oocytes while the embryonic development was ignored [34]. Additionally, in a recent meta-analysis, Hamdan et al. extracted data from 33 eligible studies, in which the synthetic results showed there were no differences in the number of oocytes retrieved between women with endometrioma who received prior surgical treatment and those who did not [35]. In general, the effect of endometrioma, especially its surgical treatment, on embryo quality, is still poorly investigated, as most existing literature focuses on its effect on oocyte quantity and quality. More research on this aspect is needed to draw a definite conclusion. The latest guideline on endometriosis developed by the European Society of Human Reproduction and Embryology (ESHRE) does not recommend the routine performance of surgery for endometrioma prior to IVF/ICSI, considering its negative impact on ovarian reserve, but it can be performed to improve the accessibility of follicles at the time of oocyte retrieval [36]. In practice, clinicians should comprehensively evaluate various clinical variables such as previous interventions, ovarian reserve, pain symptoms, bilaterality, sonographic feature of malignancy, growth, and size to determine whether the benefits of surgery for endometriomas outweigh its potential risk [37].

The strengths of this study include the large sample size and detailed baseline characteristics that enhance statistical power. Beyond that, the indicators for evaluating oocyte and embryo quality, as recommended by the Vienna consensus, are comprehensive and systematic. Currently, in other studies of this field, the indexes used to assess embryo quality do not cover the entire process of embryonic development. More importantly, the use of PSM to balance baseline differences between groups, combined with the incorporation of all variables into the propensity score model due to the large sample size, makes the analysis more statistically efficient and robust, thus reinforcing the persuasiveness of the results.

There are also some limitations to be noted in this study. Firstly, the diagnosis of endometrioma is made by ultrasonography instead of pathology, which could result in the selection bias of the study population. This is also a common limitation in the field investigating the impact of endometrioma on IVF/ICSI cycle outcomes. It is estimated that the TVUS has good accuracy for endometrioma with a sensitivity and specificity of 93% and 96%, which makes this technology more practical [38]. Secondly, the evaluation for embryo quality is based on morphological criteria in this study, however, the embryos retrieved from women with endometrioma may be intrinsically changed, which would not translate into morphological alterations. At present, the dominant laboratory performance indicators for assessing embryo quality are based on morphological features, although some other methods such as the analysis of morphokinetics by time-lapse imaging have been applied. Thirdly, the non-homogeneity of the stimulation protocols is another problem of note. Admittedly, many different stimulation protocols have been used in this retrospective study which could negatively impact the statistical data. However, the proportions of different stimulation protocols are not significantly different between the comparison groups with the use of PSM, although many different stimulation protocols have been used. Finally, both the study and control populations are confined to IVF cycles, meaning that the results cannot be extrapolated to other populations.

## 5. Conclusions

To conclude, our results suggest that endometrioma, including the corresponding surgical treatment, compromises the oocyte maturity not the embryo quality at the cleavage stage; however, surgery does not significantly influence the live birth rate, although the surgery seems to contribute to improving the blastocyst development. Furthermore, more high-quality evidence is required to elucidate the effect of endometrioma, especially its surgical treatment on embryo competence.

## Figures and Tables

**Table 1:** Baseline characteristics of the women with endometrioma and control group before and after PSM.

	Before Matching			After Matching		
Characteristic	Endometrioma Group (n = 664)	Control Group (n = 3133)	SMD	Endometrioma Group (n = 532)	Control Group (n = 532)	SMD
Age (years)	31 (28–34)	32 (29–35)	0.212	31 (29–34)	31 (28–35)	0.013
BMI (kg/m^2^)	22.33 (20.36–24.22)	23.3 (21.22–25.88)	0.367	22.5 (20.66–24.68)	22.23 (20.11–25.01)	0.024
Type of infertility						
Primary	367 (55.3)	940 (30.0)	0.508	263 (49.4)	256 (48.1)	0.026
Secondary	297 (44.7)	2193 (70.0)	0.508	269 (50.6)	276 (51.9)	0.026
Basal FSH (IU/L)	7.23 (5.99–8.91)	6.55 (5.6–7.81)	0.283	7.21 (6.00–8.55)	7 (5.91–8.48)	0.006
Basal LH (IU/L)	4.84 (3.73–6.17)	4.5 (3.36–5.88)	0.122	4.76 (3.58–5.97)	4.52 (3.40–6.09)	0.010
Basal oestradiol (pg/mL)	37.8 (27.03–52.38)	33.8 (25.72–45)	0.195	36.90 (26.05–50.78)	35.60 (25.33–49.08)	0.007
AMH	1.91 (0.92–3.75)	2.5 (1.35–4.26)	0.199	1.99 (0.98–3.88)	2.09 (1.03–3.73)	0.027
AFC	9 (6–13)	12 (9–17)	0.629	9 (7–14)	10 (7–13)	0.020
Sperm concentration (×10^6^/mL)	60.45 (40.23–84.58)	59 (39.3–86.1)	0.030	60.25 (40.33–82.5)	61.1 (38.70–85.30)	0.012
Sperm motility (%)	66.90 (56.13–79.4)	67.10 (55.9–78.3)	0.028	66.55 (56.63–78.65)	68.75 (56.90–79.10)	0.059
Sperm normal morphology (%)	5.91 (4.85–7.41)	6.00 (4.95–7.56)	0.122	5.91 (4.88–7.34)	5.91 (4.89–7.39)	0.020
Ovarian stimulation regimen						
Long GnRH agonist protocol	192 (28.9)	1512 (48.3)	0.427	188 (35.3)	186 (35.0)	0.008
Ultra-long GnRH agonist protocol	154 (23.2)	174 (5.6)	0.418	77 (14.5)	83 (15.6)	0.027
Short GnRH agonist protocol	169 (25.5)	826 (26.4)	0.021	150 (28.2)	144 (27.1)	0.026
GnRH antagonist protocol	79 (11.9)	511 (16.3)	0.136	73 (13.7)	79 (14.8)	0.035
Other	70 (10.5)	110 (3.5)	0.229	44 (8.3)	40 (7.5)	0.024
Days of ovarian stimulation	10 (9–12)	10 (9–11)	0.069	10 (9–12)	10 (9–12)	0.027
Gonadotrophin starting dose (IU)	187.5 (150–225)	150 (150–225)	0.091	175 (150–225)	175 (150–225)	0.003
Total gonadotrophin dose (IU)	2100 (1500–2913)	1800 (1356–2475)	0.194	2000 (1500–2769)	2025 (1500–2700)	0.010
Endometrial thickness on HCG trigger day (cm)	1.10 (1.00–1.25)	1.00 (0.90–1.20)	0.148	1.10 (0.95–1.25)	1.10 (0.95–1.20)	0.021
LH level on HCG trigger day (IU)	2.67 (1.43–5.08)	2.66 (1.65–4.35)	0.155	2.71 (1.52–4.82)	2.73 (1.57–4.92)	0.020
Oestradiol level on HCG trigger day (pg/mL)	2341 (1421–3425)	2825 (1802–4169)	0.266	2416 (1492–3559)	2364 (1450–3583)	0.010
Progesterone level on HCG trigger day (ng/mL)	0.81 (0.55–1.14)	0.67 (0.46–0.97)	0.136	0.80 (0.52–1.11)	0.68 (0.46–1.00)	0.035

Values are presented as median (interquartile range) or n (%). BMI: Body mass index; FSH: Follicle-stimulating hormone; LH: Luteinizing hormone; AMH: Anti-Müllerian hormone; AFC: Antral follicle count; GnRH: Gonadotropin releasing hormone; HCG: Human chorionic gonadotropin; PSM: Propensity score matching; SMD: Standardized mean difference. SMD values of less than 0.1 were considered no statistical significance.

**Table 2:** Outcomes of the women with endometrioma and control group after PSM.

Outcome	Endometrioma Group (n = 532)	Control Group (n = 532)	*p* Value
No. of oocytes retrieved	7 (4–12)	8 (4–12)	0.336
MII oocytes rate	3787/4455 (85.0)	3996/4550 (87.8)	<0.001
Normal fertilization rate	2724/4455 (61.1)	2851/4550 (62.7)	0.139
Embryo development rate on Day 2	1905/2724 (69.9)	2023/2851 (71.0)	0.402
Embryo development rate on Day 3	1061/2724 (39.0)	1121/2851 (39.3)	0.778
Good-quality embryos rate on Day 3	1482/2724 (54.4)	1601/2851 (56.2)	0.189
Blastocyst development rate	1721/2724 (63.2)	1747/2851 (61.3)	0.143
Good blastocyst development rate	1004/2724 (36.9)	1005/2851 (35.3)	0.212
Cycles cancellation rate	199/532 (27.2)	167/532 (23.9)	0.149
Clinical pregnancy rate	178/333 (53.5)	191/365 (52.3)	0.766
Miscarriage rate	21/178 (11.8)	19/191 (9.9)	0.568
Live birth rate	152/333 (45.6)	166/365 (45.5)	0.965

Values are presented as median (interquartile range) or n (%). MII: metaphase stage II; PSM: propensity score matching.

**Table 3:** Outcomes of the women with endometrioma who received surgery and the control group after PSM.

Outcome	Endometrioma with Surgery (n = 441)	Control Group (n = 441)	*p* Value
No. of oocytes retrieved	7 (4–11)	7 (4–12)	0.657
MII oocytes rate	3140/3700 (84.9)	3255/3717 (87.6)	0.001
Normal fertilization rate	2224/3700 (60.1)	2298/3717 (61.8)	0.130
Embryo development rate on Day 2	1552/2224 (69.8)	1615/2298 (70.3)	0.717
Embryo development rate on Day 3	877/2224 (39.4)	877/2298 (38.2)	0.381
Good-quality embryos rate on Day 3	1239/2224 (55.7)	1239/2298 (53.9)	0.226
Blastocyst development rate	1399/2224 (62.9)	1425/2298 (62)	0.535
Good blastocyst development rate	830/2224 (37.3)	864/2298 (37.6)	0.847
Cycles cancellation rate	161/441 (26.7)	165/441 (27.2)	0.850
Clinical pregnancy rate	148/280 (52.9)	156/276 (56.5)	0.385
Miscarriage rate	17/148 (11.5)	14/156 (9.0)	0.469
Live birth rate	125/280 (44.6)	141/276 (51.1)	0.128

Values are presented as median (interquartile range) or n (%). MII: metaphase stage II; PSM: propensity score matching.

**Table 4:** Outcomes of the endometrioma women with and without surgery after PSM.

Outcome	Endometrioma with Surgery (n = 109)	Endometrioma without Surgery (n = 109)	*p* Value
No. of oocytes retrieved	8 (5–13)	8 (5–12)	0.950
MII oocytes rate	811/1015 (79.9)	880/1003 (87.7)	<0.001
Normal fertilization rate	560/1015 (55.2)	664/1003 (66.2)	<0.001
Embryo development rate on Day 2	405/560 (72.3)	453/664 (68.2)	0.119
Embryo development rate on Day 3	237/560 (42.3)	273/664 (41.1)	0.670
Good-quality embryos rate on Day 3	310/560 (55.4)	361/664 (54.4)	0.729
Blastocyst development rate	376/560 (67.1)	400/664 (60.2)	0.013
Good blastocyst development rate	228/560 (40.7)	234/664 (35.2)	0.049
Cycles cancellation rate	44/109 (28.8)	36/109 (24.8)	0.444
Clinical pregnancy rate	33/65 (50.8)	45/73 (61.6)	0.198
Miscarriage rate	2/33 (6.1)	4/45 (8.9)	0.974
Live birth rate	30/65 (46.2)	40/73 (54.8)	0.311

Values are presented as median (interquartile range) or n (%). MII: metaphase stage II; PSM: propensity score matching.

## Data Availability

Not applicable.

## Supplementary Materials

The following supporting information can be downloaded at: https://www.mdpi.com/article/10.3390/jcm12062416/s1, Table S1: Baseline characteristics of the women with endometrioma who received surgery and the control group before and after PSM; Table S2: Baseline characteristics of the endometrioma women with and without surgery before and after PSM.
