# Supplementary material for: The Impact of Endometrioma on Embryo Quality in In Vitro Fertilization: A Retrospective Cohort Study
Source: J Clin Med. 2023 Mar 21;12(6):2416. doi: 10.3390/jcm12062416 (PMC10052961; doi:10.3390/jcm12062416)
Supplement: Supplementary file 1 [file jcm-12-02416-s001.zip › jcm-2097397-supplementary.pdf]

Table S1. Baseline characteristics of the women with endometrioma who received surgery and control group before and after PSM.

Characteristic	Before matching			After matching		
	Endometrioma with surgery (n=538)	Control group (n=3133)	SMD	Endometrioma with surgery (n=441)	Control group (n=441)	SMD
Age (years)	31 (28-33)	32 (29-35)	0.218	31 (29-34)	31 (28-34)	0.080
BMI (kg/m²)	22.33 (20.37-24.23)	23.30 (21.22-25.88)	0.356	22.43 (20.59-24.61)	22.37 (20.20-24.46)	0.053
Type of infertility						
Primary	297 (55.2)	940 (30.0)	0.507	219 (49.7)	215 (48.8)	0.018
Secondary	241 (44.8)	2193 (70.0)	0.507	222 (50.3)	226 (51.2)	0.018
Basal FSH (IU/l)	7.28 (6.06-9.09)	6.55 (5.60-7.81)	0.315	7.21 (6.05-8.59)	6.88 (5.74-8.33)	0.043
Basal LH (IU/l)	4.87 (3.75-6.19)	4.50 (3.36-5.88)	0.131	4.85 (3.62-5.99)	4.53 (3.43-5.96)	0.018
Basal oestradiol (pg/ml)	37.80 (27.22-51.85)	33.80 (25.72-45.00)	0.197	36.92 (26.65-50.40)	35.6 (26.35-49.93)	0.041
AMH	1.75 (0.85-3.36)	2.50 (1.35-4.26)	0.258	1.98 (0.98-3.62)	2.01 (0.96-3.54)	0.027
AFC	9 (6-13)	12 (9-17)	0.666	10 (7-14)	10 (7-14)	0.041
Sperm concentration ($\times 10^6$/ml)	60.10 (39.45-82.05)	59.00 (39.30-86.10)	0.054	61.10 (40.45-84.70)	58.00 (38.30-84.10)	0.034
Sperm motility (%)	67.60 (56.68-79.43)	67.10 (55.90-78.30)	0.041	67.40 (56.45-79.55)	67.60 (57.45-78.45)	0.012
Sperm normal morphology (%)	5.86 (4.81-7.34)	6 (4.95-7.56)	0.133	5.88 (4.89-7.34)	5.66 (4.93-7.18)	0.057
Ovarian stimulation regimen						
Long GnRH agonist protocol	159 (29.6)	1512 (48.3)	0.410	155 (35.1)	159 (36.1)	0.020
Ultra-long GnRH agonist protocol	106 (19.7)	174 (5.6)	0.356	63 (14.3)	69 (15.6)	0.034
Short GnRH agonist protocol	139 (25.8)	826 (26.4)	0.012	123 (27.9)	107 (24.3)	0.083
GnRH antagonist protocol	68 (12.6)	511 (16.3)	0.110	67 (15.2)	68 (15.4)	0.007
Other	66 (12.3)	110 (3.5)	0.267	33 (7.5)	38 (8.6)	0.035
Days of ovarian stimulation	10 (9-12)	10 (9-11)	0.011	10 (9-12)	10 (9-12)	0.004
Gonadotrophin starting dose (IU)	188 (150-225)	150 (150-225)	0.070	150 (150-225)	150 (150-225)	0.023
Total gonadotrophin dose (IU)	2025 (1500-2850)	1800 (1356-2475)	0.148	2000 (1500-2750)	1950 (1500-2700)	0.000
Endometrial thickness on HCG trigger day (cm)	1.10 (0.90-1.25)	1.00 (0.90-1.20)	0.088	1.10 (0.95-1.20)	1.10 (0.90-1.20)	0.031
LH level on HCG trigger day (IU)	2.81 (1.52-5.33)	2.66 (1.65-4.35)	0.184	2.67 (1.53-4.87)	2.75 (1.55-4.79)	0.014
Oestradiol level on HCG trigger day (pg/ml)	2218 (1319-3166)	2825 (1802-4169)	0.355	2386 (1515-3474)	2345 (1520-3446)	0.020
Progesterone level on HCG trigger day (ng/ml)	0.81 (0.56-1.15)	0.67 (0.46-0.97)	0.144	0.81 (0.56-1.11)	0.67 (0.46-0.97)	0.013

Values are presented as median (interquartile range) or n (%). BMI: Body mass index; FSH: Follicle-stimulating hormone; LH: Luteinizing hormone; AMH: Anti-Müllerian hormone; AFC: Antral follicle count; GnRH: Gonadotropin releasing hormone; HCG: Human chorionic gonadotropin; PSM: Propensity score matching; SMD: Standardized mean difference.

Table S2. Baseline characteristics of the endometrioma women with and without surgery before and after PSM.

Characteristic	Before matching			After matching		
	Endometrioma with surgery (n=538)	Endometrioma without surgery (n=126)	SMD	Endometrioma with surgery (n=109)	Endometrioma without surgery (n=109)	SMD
Age (years)	31 (28-33)	31 (29-34)	0.029	31 (29-35)	31 (29-34)	0.002
BMI (kg/m²)	22.33 (20.37-24.23)	22.35 (20.28-24.23)	0.047	22.04 (20.14-24.41)	22.09 (20.14-24.24)	0.048
Type of infertility						
Primary	297 (55.2)	70 (55.6)	0.007	56 (51.4)	61 (56.0)	0.092
Secondary	241 (44.8)	56 (44.4)	0.007	53 (48.6)	48 (44.0)	0.092
Basal FSH (IU/l)	7.28 (6.06-9.09)	6.74 (5.72-8.04)	0.257	7.15 (5.86-8.95)	6.82 (5.85-8.51)	0.073
Basal LH (IU/l)	4.87 (3.75-6.19)	4.66 (3.61-6.14)	0.058	5.16 (3.77-6.12)	4.74 (3.70-6.37)	0.038
Basal oestradiol (pg/ml)	37.80 (27.22-51.85)	37.90 (26.3-53.4)	0.048	36.00 (25.35-47.45)	38.00 (26.70-52.85)	0.067
AMH	1.75 (0.85-3.36)	2.40 (1.31-4.94)	0.309	2.43 (1.11-4.79)	2.35 (1.26-5.02)	0.032
AFC	9 (6-13)	9 (6-13)	0.108	9 (6-12)	9 (6-14)	0.065
Sperm concentration ($\times 10^6$/ml)	60.10 (39.45-82.05)	62.20 (42.70-91.75)	0.123	63.50 (39.95-84.05)	61.60 (42.40-89.10)	0.048
Sperm motility (%)	67.60 (56.68-79.43)	64.55 (54.38-78.78)	0.067	65.10 (53.75-79.25)	65.70 (55.45-78.85)	0.089
Sperm normal morphology (%)	5.86 (4.81-7.34)	6.09 (4.93-7.76)	0.070	5.91 (4.90-6.90)	5.99 (4.95-7.63)	0.100
Ovarian stimulation regimen						
Long GnRH agonist protocol	159 (29.6)	33 (26.2)	0.074	25 (22.9)	29 (26.6)	0.080
Ultra-long GnRH agonist protocol	106 (19.7)	48 (38.1)	0.462	40 (36.7)	37 (33.9)	0.069
Short GnRH agonist protocol	139 (25.8)	30 (23.8)	0.046	29 (26.6)	28 (25.7)	0.021
GnRH antagonist protocol	68 (12.6)	11 (8.7)	0.118	10 (9.2)	11 (10.1)	0.028
Other	66 (12.3)	4 (3.2)	0.277	5 (4.6)	4 (3.7)	0.028
Days of ovarian stimulation	10 (9-12)	11 (9-13)	0.298	10 (9-12)	11 (9-12.5)	0.097
Gonadotrophin starting dose (IU)	188 (150-225)	200 (150-225)	0.094	200 (150-225)	200 (150-225)	0.044
Total gonadotrophin dose (IU)	2025 (1500-2850)	2306 (1650-3234)	0.232	2150 (1450-3038)	2175 (1650-2962)	0.059
Endometrial thickness on HCG trigger day (cm)	1.10 (0.90-1.25)	1.20 (1.00-1.25)	0.297	1.10 (1.00-1.25)	1.10 (1.00-1.25)	0.062
LH level on HCG trigger day (IU)	2.81 (1.52-5.33)	2.37 (1.23-4.10)	0.198	2.06 (1.24-4.81)	2.51 (1.30-4.23)	0.072
Oestradiol level on HCG trigger day (pg/ml)	2218 (1319-3166)	2739 (1903-4304)	0.398	2561 (1623-3811)	2644 (1868-3957)	0.043
Progesterone level on HCG trigger day (ng/ml)	0.81 (0.56-1.15)	0.81 (0.50-1.11)	0.108	0.82 (0.57-1.17)	0.81 (0.48-1.18)	0.006

Values are presented as median (interquartile range) or n (%). BMI: Body mass index; FSH: Follicle-stimulating hormone; LH: Luteinizing hormone; AMH: Anti-Müllerian hormone; AFC: Antral follicle count; GnRH: Gonadotropin releasing hormone; HCG: Human chorionic gonadotropin; PSM: Propensity score matching; SMD: Standardized mean difference.
